# Variants in *CDH23* cause a broad spectrum of hearing loss: from non-syndromic to syndromic hearing loss as well as from congenital to age-related hearing loss

**DOI:** 10.1007/s00439-022-02431-2

**Published:** 2022-01-12

**Authors:** Shin-ichi Usami, Yuichi Isaka, Maiko Miyagawa, Shin-ya Nishio

**Affiliations:** Department of Hearing Implant Sciences, Shinshu University School of Medicine, 3-1-1 Asahi, Matsumoto, 390-8621 Japan

## Abstract

Variants in the *CDH23* gene are known to be responsible for both syndromic hearing loss (Usher syndrome type ID: USH1D) and non-syndromic hearing loss (DFNB12). Our series of studies demonstrated that *CDH23* variants cause a broad range of phenotypes of non-syndromic hearing loss (DFNB12); from congenital profound hearing loss to late-onset high-frequency-involved progressive hearing loss. In this study, based on the genetic and clinical data from more than 10,000 patients, the mutational spectrum, clinical characteristics and genotype/phenotype correlations were evaluated. The present results reconfirmed that the variants in *CDH23* are an important cause of non-syndromic sensorineural hearing loss. In addition, we showed that the mutational spectrum in the Japanese population, which is probably representative of the East Asian population in general, as well as frequent *CDH23* variants that might be due to some founder effects. The present study demonstrated *CDH23* variants cause a broad range of phenotypes, from non-syndromic to syndromic hearing loss as well as from congenital to age-related hearing loss. Genotype (variant combinations) and phenotype (association with retinal pigmentosa, onset age) are shown to be well correlated and are thought to be related to the residual function defined by the *CDH23* variants.

**Supplementary Information:**

The online version contains supplementary material available at 10.1007/s00439-022-02431-2.

## Introduction

The *CDH23* gene, a member of the cadherin superfamily, encodes calcium-dependent cell–cell adhesion glycoproteins and is known to be expressed in both the inner and outer hair cells in the cochlea. Encoded protein cadherin 23 comprises the “Tip Link” structure of the stereocilia important for hair cell function (Kazmierczak et al. 2007). Variants in the *CDH23* gene are responsible for both syndromic hearing loss (Usher syndrome type ID: USH1D) and non-syndromic hearing loss (DFNB12) (Bolz et al. 2001; Bork et al. 2001). A series of studies indicated that *CDH23* variants cause a broad range of phenotypes of non-syndromic hearing loss (DFNB12), from congenital profound hearing loss to high-frequency-involved progressive hearing loss (Wagatsuma et al. 2007; Miyagawa et al. 2012). Meanwhile, several recent studies have suggested that certain types of age-related hearing impairment (ARHI) and noise-induced hearing loss may also be associated with *CDH23* variants (Miyagawa et al. 2012; Usami et al. 2012a; Kowalski et al. 2014). Taken together, it has become clear that *CDH23* variants are likely to cause a broad range of hearing loss phenotypes.

To date, more than 190 pathologic variants have been reported for the Usher phenotype and 200 pathologic variants for the non-syndromic hearing loss phenotype (DFNB12) (Stenson et al. 2003). *CDH23*-related hearing loss has been reported in many countries with diverse ethnic backgrounds, including Cuba (Bolz et al. 2001), Germany (Bolz et al. 2001), Japan (Wagatsuma et al. 2007; Miyagawa et al. 2012; Mizutari et al. 2015), Korea (Kim et al. 2015, 2016), China (Lu et al. 2014), India (Bork et al. 2001; Ganapathy et al. 2014; Vanniya et al. 2018), Pakistan (Bork et al. 2001; Park et al. 2020), Saudi Arabia (Ramzan et al. 2020), Iran (Zardadi et al. 2020), Qatar (Alkowari et al. 2017), Turkey (Atik et al. 2015), Israel (Ashkenazi, Mizurahi, Sephardi) (Brownstein et al. 2011), Palestine (Abu Rayyan et al. 2020) and the Netherlands (Seco et al. 2017).

We have previously reported the mutational spectrum and clinical features of *CDH23*-associated hearing loss, as well as certain genotype/phenotype correlations. Meanwhile, in Japan, as genetic testing for deafness has been reimbursed by the National Health Insurance system since 2012, it has become a standard diagnostic tool for deafness. ENT clinicians can order genetic testing by application to the National Health Insurance system, allowing samples to be collected in a more unbiased manner. Currently, DNA samples as well as clinical data from more than 10,000 patients have been collected from 102 collaborative centers participating in the deafness consortium (Usami and Nishio, 2021). As the accuracy of the diagnostic strategy using massive parallel sequencing (MPS) has improved over the last few years, this study was conducted using large-cohort data to revise the mutational spectrum of *CDH23* as well as to verify if there are certain genotypes correlated with such a wide range of phenotypes.

## Subjects and methods

### Subjects

For this study, a total of 12,139 Japanese hearing loss patients and controls (autosomal dominant sensorineural hearing loss; ADSNHL, 2462; autosomal recessive sensorineural hearing loss; ARSNHL or sporadic, 6912; inheritance unknown, 2220; unilateral hearing loss, 212; and normal hearing control subjects, 333) were recruited from 102 otolaryngology departments nationwide. Among these subjects, we selected patients with CDH23 variants based on MPS for 63 target genes. Prior to participation in this study, which was approved by the Shinshu University Ethical Committee and the ethical committee within each participating institution, written informed consent was obtained from all patients (or from their next of kin, caretaker or guardian in case of minors or children). Clinical information and peripheral blood samples were obtained from each subject and from all their consenting relatives. This study was conducted in accordance with the Declaration of Helsinki, and the protocol was approved by the Ethics Committee of Shinshu University School of Medicine (No. 387-4 September 2012 and No. 576-2 May 2017).

### MPS analysis

Sixty-three genes (Nishio et al. 2015) reported to be causative of non-syndromic hearing loss (Hereditary Hearing loss Homepage; http://hereditaryhearingloss.org/) were analyzed in this study. The detailed protocols for targeted enrichment and DNA sequencing have been described elsewhere (Nishio et al. 2015). In brief, amplicon libraries were prepared using the Ion AmpliSeq Custom Panel, with the Ion AmpliSeq Library Kit 2.0 and the Ion Xpress Barcode Adapter 1–96 Kit (Life Technologies) according to the manufacturer’s instructions. After the amplicon libraries were prepared, equal amounts of the libraries for six patients were pooled for one Ion PGM sequence reaction and those for 45 patients were pooled for one Ion Proton system sequence reaction with an Ion P1 chip or an Ion S5 system sequence reaction with an Ion 540 chip according to the manufacturer’s instructions. The sequence data were mapped against the human genome sequence (build GRCh37/ hg19) with the Torrent Mapping Alignment Program. Subsequently, DNA variants were piled up with the Torrent Variant Caller plug-in software included in the Torrent Suit (Life Technologies). After variant detection, the effects of the variants were analyzed using ANNOVAR software (Wang et al. 2010). The missense, nonsense, insertion/deletion and splicing variants were selected among the identified variants. Variants were further selected from < 1% of several control databases including the 1000 genome database (http://www.1000genomes.org/), the 6,500 exome variants (http://evs.gs.washington.edu/EVS/), The Genome Aggregation Database (https://gnomad.broadinstitute.org), the human genetic variation database (dataset for 1208 Japanese exome variants) (http://www.genome.med.kyoti-u.ac.jp/SnpDB/index.html), the 8300 Japanese genome variation database (https://jmorp.megabank.tohoku.ac.jp/202102/) and the 333 in-house Japanese normal hearing controls. The filtering procedures were performed using our original database software as described previously (Nishio and Usami 2017). The pathogenicity of the identified variants was evaluated in accordance with the American College of Medical Genetics (ACMG) standards and guidelines (Richards et al. 2015) with ClinGen hearing loss clinical domain working group expert specification (Oza et al. 2018). To validate the identified variant, Sanger sequencing analysis was performed using PCR and exon-specific custom primers according to the manufacturer’s instructions. All primers were designed using the web version of Primer 3 plus software (http://www.bioinformatics.nl/cgi-bin/primer3plus/primer3plus.cgi).

## Results

**Fig. 1:**
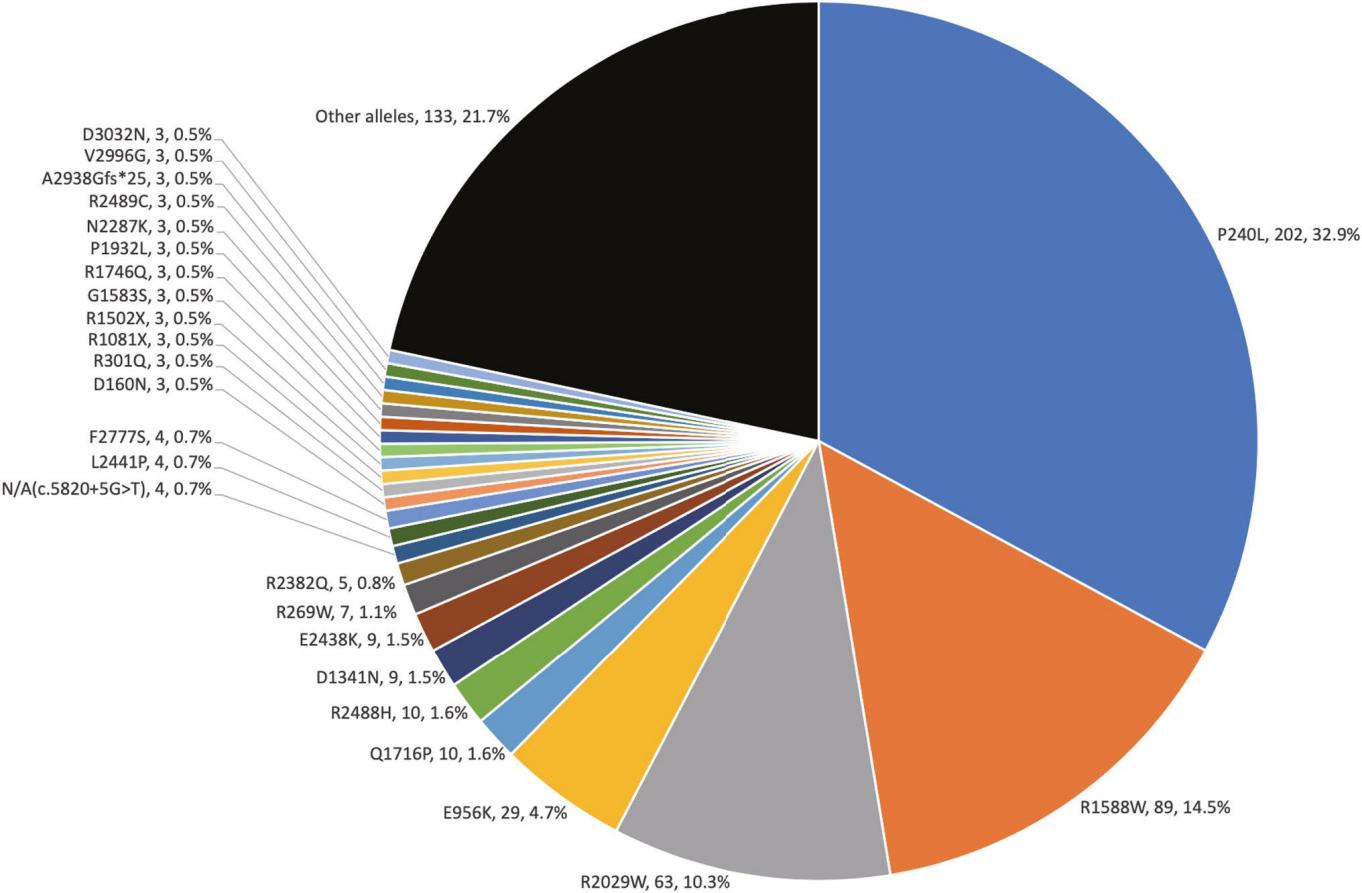
*CDH23* variants identified in this study. A total of 126 possible disease-causing *CDH23* variants were identified. The frequently found variants (p.P240L (32.9%), p.R1588W (14.5%), p.R2029W (10.3%) and p.E956K 4.7%) accounted for 62.4% of the mutations

As a result of the large-cohort MPS analysis, we identified 307 probands with *CDH23*-associated hearing loss. Among the 307 probands, 29 were identified from ADSNHL or maternally inherited cases, whereas 273 were identified from ARSNHL or sporadic cases, with no familial information available for five cases. A total of 126 possibly disease-causing *CDH23* variants were identified, 37 of which were previously reported and 89 were novel (Supplementary Table 1). The variants consisted of 90 missense variants, 9 nonsense variants, 11 splicing variants and 16 frameshift deletion variants. Variants were defined as likely causative variants if the following criteria were fulfilled: (1) pathogenic or likely pathogenic based on the ACMG criteria, or (2) in the case of variants of uncertain significance (VUS) based on the ACMG criteria, where significant CADD scores (> 20) were observed, (3) biallelic variants found in recessive inheritance cases, (4) no other candidate variants were found and (5) there was no contradiction with the family analysis (if samples from family member were available). Based on the ACMG guidelines, the variants were categorized into 24 pathogenic, 40 likely pathogenic and 62 VUS. Among the mutations, p.P240L was the most common (32.9%; 202/614 allele), followed by p.R1588W (14.5%; 89/614 allele), p.R2029W (10.3%; 63/614 allele) and p.E956K (4.7%; 29/614 allele) (Fig. 1). The minor allele frequencies (MAFs) of these four highly prevalent variants in the Japanese and other ethnic groups are shown in Supplementary Table 1. The MAFs in the Japanese population are significantly higher than those in the other ethnic groups. Fourteen variants (4 out of 24 pathogenic variants, 4 out of 40 likely pathogenic variants and 6 out of 62 VUS) were found in the DRE, DXNDN, or DXD motif (Supplementary Table 1).

Various combinations of biallelic variants were detected and a total of 307 subjects were diagnosed as suffering hearing loss caused by *CDH23* variants (Supplementary Table 2). Among them, six patients had associated visual impairment (Supplementary Table 3).

**Fig. 2:**
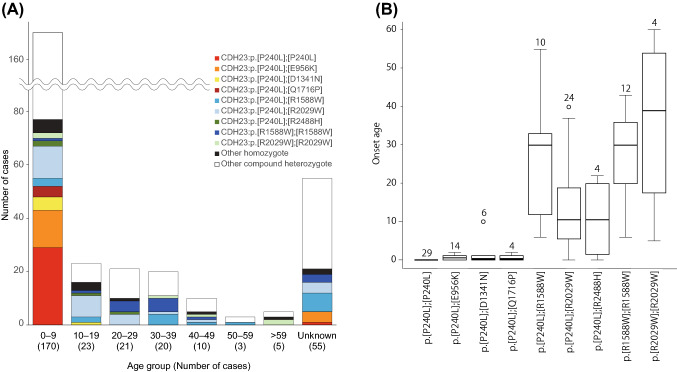
Age of onset and combination of *CDH23* variants.** A** The age of onset and the combination of variants were closely related. Most of the patients with p. [P240L] or p. [E956K] showed congenital/early onset, while the patients with p.[R1588W] or p. [R2029W] showed late-onset hearing loss. **B** Frequently found combinations of *CDH23* variants and onset age. The combinations p.[P240L];[P240L], p.[P240L];[E956K], p.[P240L];[D1347N] and p.[P240L];[Q1716P] showed congenital onset, whereas patients with p.[P240L];[R1588W], p.[P240L];[R2029W], p.[P240L];[R2488H], p.[R1588W];[R1588W] and p.[R2029W];[R2029W] showed late-onset hearing loss. The number on each box indicates the number of patients. The error bar on each box indicates standard deviation

As shown in Fig. 2A, an overall histogram of onset age was compiled for the congenital/early-onset hearing loss population. It should be noted that a small but significant number of patients showed late-onset (from the 2nd to the 7th decade) hearing loss. The age of onset seemed to be associated with specific variant combinations; i.e., the patients with p.[P240L];[P240L], p.[P240L];[E956K], p.[P240L];[D1347N] and p.[P240L];[Q1716P] showed congenital onset, whereas those with p.[P240L];[R1588W], p.[P240L];[R2029W], p.[P240L];[R2488H], p.[R1588W];[R1588W] and p.[R2029W];[R2029W] showed late-onset hearing loss (Fig. 2A, B) .

**Fig. 3:**
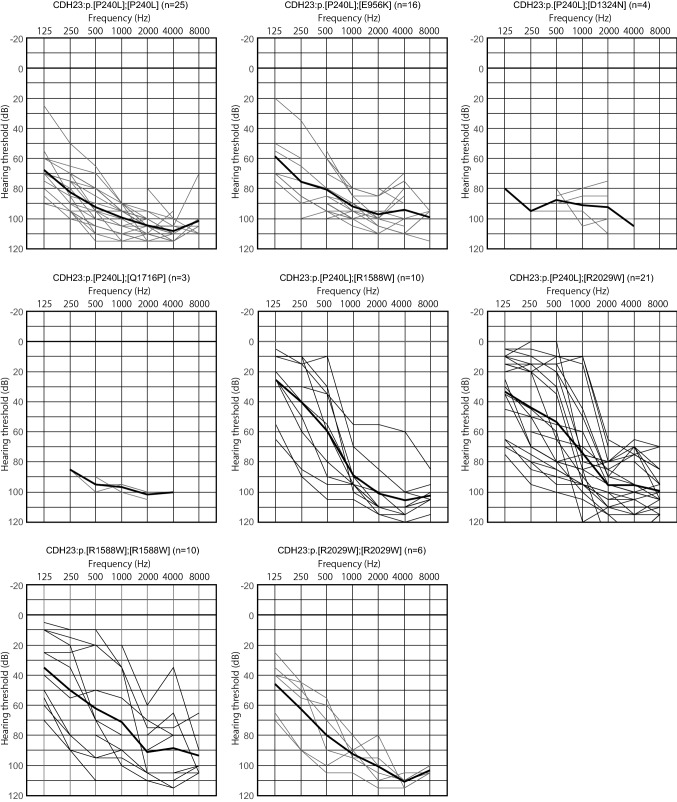
Genotype and audiogram configurations. With regard to genotype/phenotype correlations, in contrast to the patients with p.[P240L];[P240L], p.[P240L];[E956K] p.[P240L];[D1347N] and p.[P240L];[Q1716P] who exhibited more severe hearing loss with limited residual hearing in the lower frequencies, the majority of patients with p.[P240L];[R1588W], p.[P240L];[R2029W], p.[R1588W];[R1588W] and p.[R2029W];[R2029W] showed high-frequency-involved hearing loss. The bold lines indicate the average hearing threshold for each genotype

With regard to genotype/phenotype correlations, in contrast to the patients with p.[P240L];[P240L], p.[P240L];[E956K] p.[P240L];[D1347N] and p.[P240L];[Q1716P] who exhibited more severe hearing loss with limited residual hearing in the lower frequencies, the majority of patients with p.[P240L];[R1588W], p.[P240L];[R2029W], p.[R1588W];[R1588W] and p.[R2029W];[R2029W] showed high-frequency-involved hearing loss (Fig. 3).

**Fig. 4:**
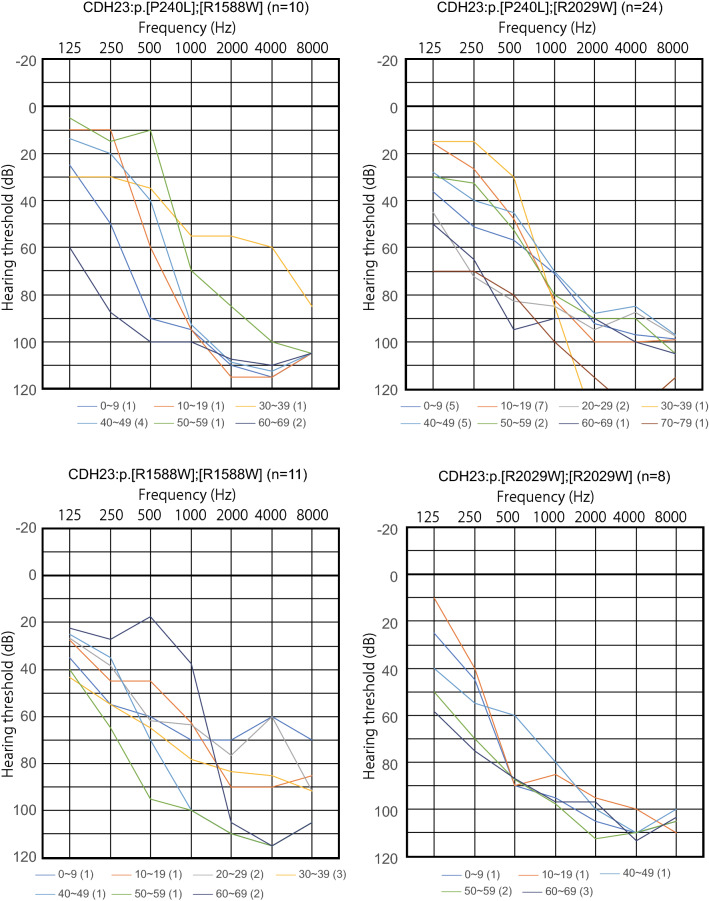
Progression of hearing loss in patients with *CDH23* variants. The figure shows the hearing level for each age group. The patients with the four variant combinations involving p.[R1588W] or p. [R2029W] showed progressive hearing loss

Progression was analyzed in patients with residual hearing with four combinations of biallelic variants; p.[P240L];[R1588W], p.[P240L];[R2029W], p.[R1588W];[R1588W] and p.[R2029W];[R2029W] (Fig. 4). The patients with any one of these four combinations of variants showed progressive hearing loss (Fig. 4).

Of the 198 cases for which clinical data were available, 59 used hearing aids (HAs) and 120 used cochlear implants or electric acoustic stimulation (CI/EAS), with 13 cases not wearing any aids and 6 unknown cases (Supplementary Table 2).

**Fig. 5:**
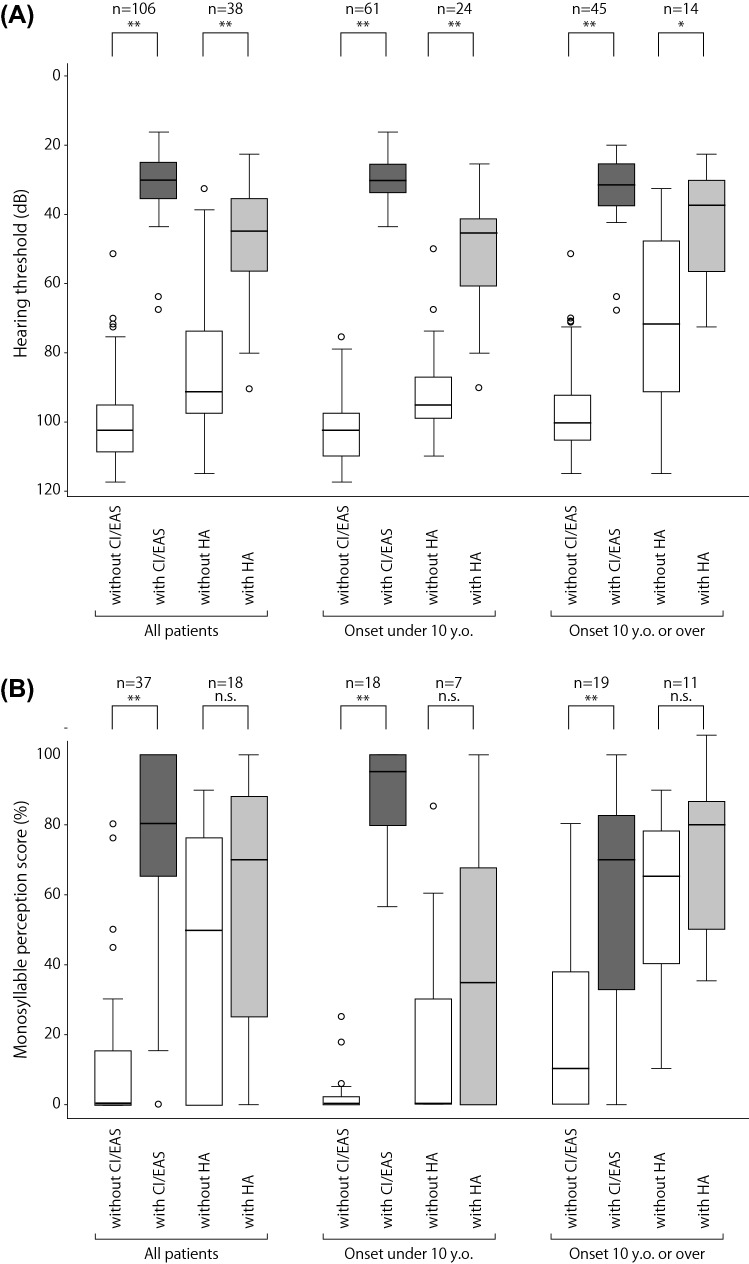
Benefits of HAs and/or CI/EAS. HAs and/or CI/EAS show good outcomes for patients with *CDH23* variants. **A** Hearing threshold of the patients with/without HA or CI/EAS. **B** Monosyllable perception score of the patients with/without HA or CI/EAS. The corresponding two groups were tested by *t* test. The number on each group indicates the number of patients. *p < 0.05, ** *p* < 0.01

With regard to the effects of hearing aids and cochlear implantation, hearing threshold (dB) and monosyllable perception score (%) were found to be significantly improved after these interventions (Fig. 5).

## Discussion

### Frequency

As described in “Results”, through the screening for 63 deafness genes in this study, we focused on patients with *CDH23* variants. No other candidate variants were found in these patients. The present large-cohort study revealed that the prevalence of *CDH23*-associated hearing loss was 2.64% (307/11,594) among bilateral SNHL probands and 3.95% (273/6912) among ARSNHL/sporadic probands in this Japanese population. Some patients (*n *= 29) were found in ADSNHL families, which is probably due to a pseudo-dominant inheritance pattern. These frequencies are slightly higher than those in our previous report (1.6% in total, 2.5% in ARSNHL) (Miyagawa et al. 2012), but this is due to differences in the methodology; this study used sequencing for the entire exons of the *CDH23* gene in contrast with the previous screening based on TaqMan for a limited number of common variants. Based on the use of over 10,000 samples collected in a more unbiased manner, the present study indicated the prevalence of *CDH23*-associated hearing loss among non-syndromic SNHL patients. *CDH23*-associated hearing loss, along with *SLC26A4*, is the second or third most frequent type of hearing loss after *GJB2*-associated hearing loss. (Nishio and Usami 2015; Usami and Nishio, 2021), and *CDH23* variants are an important cause of non-syndromic SNHL. However, hearing loss due to *CDH23* variants is a frequent cause of hearing loss, not only in the Japanese population, but also in many ethnicities, such as Netherlanders, Palestinians, Egyptians, and Jews (see Usami and Nishio 2021 for review).

### Mutational spectrum

The present study demonstrated a total of 126 possible disease-causing *CDH23* variants, including 37 previously reported and 89 novel variants (Supplementary Table 1). As in our previous reports on DFNB12 (Miyagawa et al. 2012), a majority of variants was found in the EC domain, with only a few exceptions found in the cytoplasmic domain. Fourteen out of the 126 possible causative variants were found in the DRE, DXNDN and DXD motifs, which are thought to be important for calcium binding. These highly conserved EC calcium-binding motifs are thought to be essential for linearization, rigidification, and dimerization of the cadherin molecules (Nagar et al. 1996; Angst et al. 2001). It should be noted that p.E956K is located in the DRE motif, which is in agreement with the comparatively severe DFNB12 phenotype. According to recent computer analysis for the prediction of the impact of amino acid changes to protein structures, some possible pathologic variants are predicted to cause severe damage to the protein function of cadherine 23 (Supplementary Fig. 1).

This study revealed that there are several common variants in the Japanese hearing loss population, with p.P240L accounting for 32.9% of all *CDH23* variants in the Japanese population, followed by p.R1588W (14.5%), p.R2029W (10.3%) and p.E956K (4.7%). These four common variants account for 62.4% of all variants. For such recurrent variants, founder effects have been demonstrated in many deafness genes: for example, with regard to *GJB2*, it is reported that c.35delG, which is predominant throughout Europe, the Middle East, North Africa, North and South America and Australia; and c.235delC, which is commonly found in East Asians, are due to founder effects (see review; Tsukada et al. 2015). The p.P240L variant in the *CDH23* gene, the most frequent variant in the Japanese as well as the Korean population, has been proven to be due to a founder effect using the STR marker (Kim et al. 2015). In fact, the MAF in Japanese controls is exceptionally high compared to those in other ethnic groups (Supplementary Table 1), which is consistent with the fact that there are many patients with *CDH23*-related hearing loss due to the founder effect.

### DFNB12 phenotype vs. Usher phenotype

In this study, we were able to collect current clinical data for approximately two-thirds (198/307) of the patients. According to the age at which clinical information was collected (see Supplementary Table 2) and the age range of patients with each combination of biallelic variants, visual symptoms were not observed, despite the inclusion of a large number of patients aged 10 years and older. Phenotype classification (DFNB12 or Usher phenotype) is shown in Supplementary Table 2. It is necessary to take into consideration that patients with “non-syndromic (NS)” hearing loss up to the second decade may experience visual symptoms in the future. However, some cases with the same variant do not present with visual symptoms even in the third decade. For example, in terms of p. [P240L]; [P240L], 22 patients were within the second decade, so the term NVS (no visual symptom to date) is used; however, as no visual symptoms were observed in two patients over the third decade, it was speculated that this variant shows a non-syndromic phenotype. The same results can be observed for many variants. The majority of patients (192/198) were found to not have any associated visual symptoms; therefore, most patients with the *CDH23* variants are likely to present with a non-syndromic phenotype.

This finding seems to be related to the fact that the majority of the causative variants (either pathogenic, likely pathogenic or VUS) identified in this study (Supplementary Table 1) were missense mutations, which are supposed to have a residual function. With regard to genotype/phenotype correlations, the DFNB12 phenotype is reported to be associated with biallelic missense mutations, whereas the USH1D phenotype is associated with presumably functional null alleles, including nonsense, splice-site, frameshift or some missense mutations (Bork et al. 2001; Wagatsuma et al. 2007; Oshima et al. 2008; Miyagawa et al 2012). It has been reported that cases in which an Usher allele and DFNB12 allele are present in the *trans* configuration show a non-syndromic phenotype (Schultz et al. 2011). In this study, No. 3330 with a biallelic homozygous missense mutation (p. [R2489C]; [R2489C]) showed a USH phenotype, whereas p. [R1588W]; [R2489C] and p. [Y2301H]; [R2489C], which had a compound heterozygous HL, showed a non-syndromic phenotype (Supplementary Table 2). In this study, the majority of patients with biallelic missense mutations or those with compound heterozygous missense and truncating mutations showed the DFNB12 phenotype, which is generally in line with this rule. In our cohort, a limited number of patients (6/307) were found to show the Usher phenotype (Supplementary Table 3). Five out of six cases were associated with at least one truncating mutation and had visual impairment (Usher phenotype), which also supports this rule. However, patient No. 3330 with a biallelic homozygous missense mutation (p.[R2489C]; [R2489C]) and patient No. 4177 with a nonsense mutation and missense mutation (p.[Y288X]; [G2017S]) also showed the Usher phenotype. Although the functional significance of these missense mutations is unknown, we presume that they are functionally null alleles based on the phenotypes identified herein. Therefore, future functional analysis will be necessary to resolve this inconsistency. When discussing genotype–phenotype correlations, we must take into account that there are always exceptions.

Of course, it is important to pay attention to the visual symptoms during the follow-up period, but other clinical symptoms predicting the Usher phenotype include a delay in starting to walk. USH1D is known to be associated with vestibular dysfunction. Among six Usher phenotype patients in this study, only one patient (No. 3687 in Supplementary Table 3) had undergone vestibular function testing. The patient had no bilateral response on the caloric test. In this study, instead, we collected clinical information, thinking that “the month at which the head is supported when sitting” and “the month at which walking started” could be indirect evidence of a developmental delay and vestibular dysfunction. As a result, it was clarified that five out of six patients with Usher syndrome had a delayed start to walking, which could be indirect proof of vestibular dysfunction (Supplementary Table 2). This was also supported by the fact that most of the patients had no delay in starting to walk; that is, there were not many cases of *CDH23*-related hearing loss with the Usher phenotype, as inferred from the clinical data. Although it is difficult to evaluate the vestibular function in young children, it may be possible to identify the USH phenotype more accurately by performing pediatric vestibular assessments (Dhondt et al. 2018).

### A wide range of hearing loss: clinical characteristics and genotype/phenotype correlations

Based on the data for more than 10,000 hearing loss patients, the present updated study clearly demonstrated that *CDH23* variants cause a wide range of hearing loss from non-syndromic hearing loss (DFNB12) to syndromic hearing loss and Usher syndrome type ID (USH1D). Also, the present results showed that most cases of *CDH23*-associated hearing loss are congenital/early onset. Nonetheless, a certain number of cases of late-onset (up to the 60 s) progressive hearing loss were also identified. As shown in Fig. 2A, B, a wide range of onset ages (awareness of hearing loss) was found, ranging from congenital to 60 + years old, although the majority of cases were congenital or early onset. Genotype (variant combinations) and phenotype (onset age) were shown to be well correlated. The patients with p.[P240L];[P240L] and p.[P240L];[E956K] showed congenital and severe hearing loss, whereas the patients with the p.R2029W or p.R1588W variant showed late-onset high-frequency-involved hearing loss (Figs. 2A, B, 3). We have previously reported the clinical characteristics of *CDH23*-related hearing loss to be high-frequency-involved progressive hearing loss (Miyagawa et al. 2012). With regard to audiogram configurations, the majority of patients had some residual hearing in the lower frequencies, as reported previously (Wagatsuma et al. 2007; Miyagawa et al. 2012). Further, the progressive nature of the hearing loss was demonstrated by serial audiograms (Miyagawa et al. 2012), and reconfirmed using audiograms with the average for each age plotted (Fig. 4). To date, several replication studies stating the same clinical features have been reported (Mizutari et al. 2015; Kim et al. 2016; Ramzan et al. 2020). Combined with these reports, the present large-cohort data confirmed that this clinical feature tends to be constant regardless of the type of variant.

**Fig. 6:**
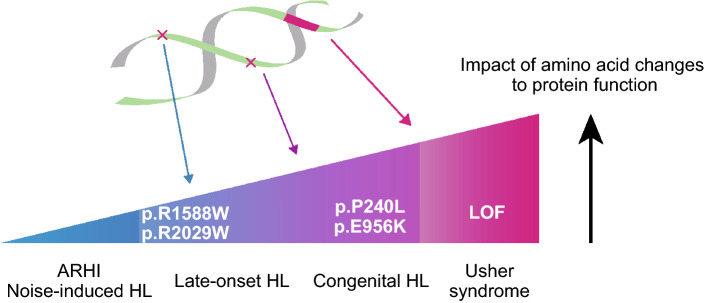
Image of genotype/phenotype correlations: it is presumed that various phenotypes are caused by the residual function of the cadherine 23 protein

In addition, we have previously reported some types of ARHI due to *CDH23* variants, both of which had hearing loss due to a homozygous mutation in p.R2029W (Usami et al. 2012a). Such a late-onset phenotype is not surprising as a series of animal studies have shown that *CDH23* variants are involved in the C57BL/6 mouse strain, which is the most common mouse model for ARHI (Noben-Trauth et al. 2003). ARHI is believed to be a typical complex disorder associated with both genetic factors and environmental factors. Degeneration and age-related changes in the cochlea might be accelerated by accumulated external and internal factors. These environmental factors, including exposure to noise, ear disease, ototoxic drugs and associated disease (circulatory disease, diabetes mellitus, etc.) play important causative roles in ARHI. Therefore, in addition to the monogenically inherited particular type of ARHI shown in this study, various SNPs may be involved in susceptibility to ARHI. It should be noted that variants of *CDH23* are reported to be associated with noise-induced hearing loss (Kowalski et al. 2014) (Fig. 5). Taken together, these findings suggest that the residual function defined by the *CDH23* variants can cause various types of hearing loss, from non-syndromic to syndromic hearing loss as well as from congenital to age-related hearing loss (Fig. 6).

### Intervention perspective

In this study, 198 patients for whom clinical data concerning interventions were available used HAs or CI/EAS, indicating that these are common therapeutic interventions used in Japan. Figure 5 clearly shows the benefits of HAs and/or CI/EAS. In terms of CI/EAS, our series of papers demonstrated that CI is a good therapeutic option for patients with hearing loss over all frequencies, and EAS is a good option for patients with residual hearing (Usami et al. 2012b, 2020; Miyagawa et al. 2013; Moteki et al. 2017, 2018; Yoshimura et al. 2020). As a significant portion of patients with *CDH23* variants have residual hearing, it is extremely important to perform atraumatic CI surgery to preserve residual hearing for this particular category of patients. With regard to post-operative residual hearing after EAS, we have demonstrated that the hearing preservation rate among patients with mutations in stereocilia-related genes, such as *CDH23*, *MYO7A*, or *MYO15A*, was statistically better compared to the patients with other etiologies (Yoshimura et al. 2020). With regard to the progression of hearing loss in patients with *CDH23* mutations, it is better to use long electrodes for CI/EAS that can cover low-frequency region (Usami et al. 2020). Genetic testing is also useful for estimating the presence of residual hearing for very young children for whom residual hearing is difficult to measure by auditory brainstem response (ABR) (Usami et al. 2012b).

## Conclusion

This paper clearly shows the mutational spectrum of *CDH23* in Japanese hearing loss patients as well as genotype–phenotype correlations revealed through genetic analysis using more than 10,000 patients. In the present large-cohort study, variants in *CDH23* were shown to cause a broad spectrum of hearing loss: from non-syndromic to syndromic hearing loss as well as from congenital to age-related hearing loss. Although these results are based solely on the analysis of Japanese hearing loss patients, the fundamental rule is believed to be the same regardless of racial differences.

## Supplementary Information

Below is the link to the electronic supplementary material.

Supplementary file1 (XLSX 25 KB) Table 1 *CDH23* variants identified in this study. The minor allele frequencies (MAFs) of variants in the Japanese and other ethnic groups are shown.

Supplementary file2 (XLSX 21 KB) Table 2 Combinations of biallelic *CDH23* variants detected and the hearing devices applied in this study

Supplementary file3 (XLSX 12 KB) Table 3 Combinations of biallelic *CDH23* variants showing associated visual impairment

Supplementary file4 (EPS 4897 KB) Figure 1: Three-dimensional modeling of WT cadherin 23 and the p.E956K variant. A 3-dimensional model was built by comparative modeling using the SWISS-MODEL workspace and with the coordinates of human *CDH23* (PDB accession number 3q2w) as a structural template. The results of the 3-dimensional analysis were visualized by MacPyMOL version 0.99rc.6 (http://www.pymol.org)
